# Response surface optimization of process parameters for preparation of cellulose nanocrystal stabilized nanosulphur suspension

**DOI:** 10.1038/s41598-023-47164-y

**Published:** 2023-11-24

**Authors:** Manoj Kumar Mahawar, Ashok Kumar Bharimalla, A. Arputharaj, Jagdish Palkar, Jyoti Dhakane-Lad, Kirti Jalgaonkar, N. Vigneshwaran

**Affiliations:** ICAR-Central Institute for Research on Cotton Technology, Mumbai, 400019 India

**Keywords:** Other nanotechnology, Techniques and instrumentation

## Abstract

This study employed response surface methodology (RSM) to optimize various parameters involved in the synthesis of nanosulphur (NS) stabilized by cellulose nanocrystals (CNCs). The elemental sulphur (ES) mixed with CNCs was processed in a high-pressure homogenizer to make a stable formulation of CNC-stabilized NS (CNC-NS). RSM was adopted to formulate the experiments using Box-Behnken design (BBD) by considering three independent variables i.e., ES (5, 10, 15 g), CNCs (25, 50, 75 ml), and the number of passes (NP) in the high-pressure homogenizer (1, 2, 3). For the prepared suspensions (CNC-NS), the range of the responses viz. settling time (0.84–20.60 min), particle size (500.41–1432.62 nm), viscosity (29.20–420.60 cP), and surface tension (60.35–73.61 N/m) were observed. The numerical optimization technique was followed by keeping the independent and dependent factors in the range yielded in the optimized solution viz. 46 ml (CNCs), 8 g (ES), and 2 (NP). It was interpreted from the findings that the stability of the suspension had a positive correlation with the amount of CNC while the increasing proportion of ES resulted in reduced stability. The quadratic model was fitted adequately to all the responses as justified with the higher coefficient of determination (R^2^ ≥ 0.88). The characterization performed by X-ray diffraction (XRD), zeta potential, Raman spectroscopy, and Fourier transform infrared spectroscopy (FTIR) revealed better-stabilizing properties of the optimized CNCs–ES suspension. The study confirmed that CNCs have the potential to be utilized as a stabilizing agent in synthesizing stable nanosulphur formulation by high-pressure homogenization.

Sulphur is one of the most abundant elements in the universe, and is among the prime by-products of many petrochemical industries^1^. Sulphur being a reactive non-metal has drawn attention recently due to its abundance, and a variety of functionalities in different industries including sulphuric acid production, agriculture, plastics, chemical industry, pulp and paper, rubber industry, and other agrochemical industries^2^. The global sulphur production in 2020–21 was around 80 million tonnes, while India's sulphur production was 3.5 million tonnes^3^. The fertilizer industry is the largest consumer of sulphur, accounting for about 50–60% of sulphur demand^4^. But, the industrial and agricultural application of elemental sulphur (ES) is associated with several limitations due to its micron size range. Size reduction is very much essential to make it readily available to the plant in case of fertilizer use and for faster reactivity in industrial applications.

Nanotechnology is an established and novel scientific approach capable of manipulating materials by modifying their physical and chemical properties at molecular levels. The nanomaterials have smaller size, increased surface area, better stability, and higher reactivity as compared to their bulk counterparts^5^. Cellulose is a straight-chain polymer of numerous d-glucose units linked together in a reiterating, overlapping pattern, resulting in a high tensile strength polymer. It is the main structural component of the primary cell wall of plants, many forms of algae, and fungi. For industrial use, cellulose is obtained from wood pulp, agro-biomass, and cotton^6^. Nanocellulose is a novel biomaterial derived from any cellulosic biomass by various processes viz., mechanical, chemical, biological, and combinations. Renewable nature, anisotropic shape, excellent mechanical properties, good biocompatibility, surface functionality, hydrophilicity, remarkable optical properties with self-assembling capability, excellent tensile strength, lack of toxicity, and biodegradability make nanocellulose an efficient stabilizing agent for emulsions^7,8^.

The reported literature demonstrated that owing to its low cost, easy availability, safe, green, natural, and readily accessible for physical or chemical modifications, nanocellulose has the potential of a good stabilizing agent for various applications^9–11^. Cieśla et al.^12^ demonstrated the use of nanocellulose from carrot pomace as stabilizer in the synthesis of silver nanoparticles. The authors reported the interaction between carboxylic groups on the surface of cellulose with silver nanoparticles and the spatial structure on the surface of nanocellulose as important reasons for its stabilizing property. Similarly, Teo et al.^13^ described the utilization of nanocellulose derived from plants as stabilizer in pickering emulsion. Liu et al.^14^ derived nanocellulose from corn stover and subsequent to surface modification has improved the freeze–thaw stability of oil-in-water. Wang et al.^15^ High-pressure homogenization is an established technique for size reduction where the samples were passed through a narrow nozzle at high velocity and pressure in the range of 100–4200 kg/cm^2^.

The review of literature lacked any published report of utilizing CNC for the stabilization of sulphur nanoparticles. Therefore, the authors in this study attempted to substantiate the stabilization attribute of CNC which resulted in a novel and eco-friendly sulphur based suspension. It can be reiterated that owing to the surface chemistry, aspect ratio, and crystallinity, CNCs provide stability to the dispersions as a possible alternative for commercial surfactants.

Response surface methodology (RSM) has been successfully applied as an empirical statistical technique for multifactor regression analysis and optimization studies concerning a variety of problems. The generated regression equations of the responses are evaluated using RSM and help in identifying the optimum values of the process variables^16^. Box-Behnken design (BBD) was adopted by taking three independent variables i.e., ES, CNCs, and NP to optimize the process for the production of stable suspension of CNC stabilized NS (CNC-NS). Keeping the above facts in consideration, objective of the present study was framed to process ES in combination with CNCs through high-pressure homogenization to achieve stable nanosulphur formulation. The optimization of the process parameters using RSM was also investigated.

## Experimental

### Material

The ES powder having a particle size distribution in the range of 200–250 microns was procured from the local market of Mumbai, Maharashtra (India). Linters from commercial cotton cultivars available at ICAR-Central Institute for Research on Cotton Technology, Mumbai, were used for the production of CNCs. All other chemical reagents were of analytical grade and the water was deionized.

### Preparation of CNCs

Cotton linters were kier boiled and bleached followed by acid hydrolysis to form microcrystalline cellulose that served as a precursor for CNCs preparation. Microcrystalline cellulose was pre-treated with cellulase enzyme (1% concentration) at 45 °C for 30 min in stirred condition. After the removal of the cellulase enzyme by filtration, the material was passed through a high-pressure homogenizer (Microfluidics®, Model: M-700, USA) operated at 1800 kg/cm^2^ pressure for the first pass and 2100 kg/cm^2^ for the remaining passes^17^. The suspension was lyophilized (− 80 °C for 48 h) using freeze dryer (M/s SP Scientific, Model: Virtis Virtual 50 XL, USA) further to estimate the concentration of CNCs.

### CNCs’ characterization

The CNCs’ was evaluated for particle size using size analyser (M/s Cordouan Technologies®, France, scattering angle: 170°) and zeta potential (M/s Nicomp™ 380 ZLS particle size analyser) by electrophoretic light scattering principle.

### Experimental design

Response surface methodology (Design expert®, Version 13, Stat Ease, Minneapolis) was used to design the experiments. Based on the preliminary trials, the range of the independent variables i.e., CNCs (A) (25, 50, 75 ml), ES (B) (5, 10, 15 g), and the NP (C) (1, 2, 3) in homogenizer was selected. The sample volume was made up to 100 ml using deionized water for all the runs. Box Behnken design was selected and the total number of experiments was 17 including 5 central point experiments. The coded and actual levels of the independent variables are given in Table 1.

**Table 1:** BBD matrix for the experimental run with actual and coded values (in parenthesis).

Independent variables	Coded (in parenthesis) and actual levels		
	(− 1)	(0)	(1)
Cellulose nanocrystals (CNCs), ml (A)	25	50	75
Elemental sulphur (ES), g (B)	5	10	15
Number of passes, (NP), (C)	1	2	3

### Preparation of CNC-NS

The independent variables were varied accordingly as per the experimental design to obtain different suspensions. The mixture of CNCs and ES was stirred using a rotary stirrer (M/s Remi, 1/8 HP, India) for 15 min to ensure uniform mixing before feeding to the homogenizer. The laboratory model high-pressure homogenizer (M/s Stansted® Homogenizing Systems Ltd, Model S-PCH-10, UK) was operated at a pressure of 2100 kg/cm^2^. The valve-type homogenizer was used to avoid possible blockage during the operation. The homogenizing valve of this system features a ceramic needle and seat with profiled geometry for optimal performance. Adequate amounts of ES and CNCs were pre-mixed and homogenized for one, two, and three passes accordingly, as per the BBD matrix. The samples were collected for analysis and the machine was flushed with deionized water after each run to avoid any contamination.

### Statistical analysis

The quadratic model was fitted to the obtained responses and analysis of variance (ANOVA) was performed. The coefficient of determination (R^2^), adjusted R^2^, coefficient of variation (CV), lack of fit, etc. were considered for evaluating the model significance. *p* value less than 0.05 indicated that the model terms were significant, and values greater than 0.10 indicated the model terms were not significant. The predicted response variables (Y) were analyzed by the following second-order polynomial regression shown in the Eq. 1:

1 $$Y={a}_{0}+{a}_{1}{x}_{1}+{a}_{2}{x}_{2}+{a}_{3}{x}_{3}+{a}_{11}{x}_{12}+{a}_{22 }{x}_{2}^{2}+{a}_{33}{x}_{3}^{2} +{a}_{12}{x}_{1}{x}_{2}+{a}_{13}{x}_{1}{x}_{3}+{a}_{23}{x}_{2}{x}_{3}$$

where Y is the predicted response variables, a_0_ is the intercept terms, x_i_ is the independent factor coded/actual terms, a_i_ is the model coefficient terms.

### Optimization

The Box–Behnken design involves three equal levels (low (− 1), medium (0), and high (+ 1)) of the independent variables with a lesser number of experiments. Three independent variables were optimized using BBD-based RSM optimization technique employing Design Expert software. The optimization was performed by setting goals for the independent and dependent variables i.e., by keeping the independent parameters and the responses viz. particle size, viscosity, settling time, and surface tension in range. The relative effect of the process variables on the responses was studied to optimize the process variables.

### Characterization of the suspension

#### Settling time

The settling time (ST) was evaluated by keeping 100 ml of suspension in a graduated measuring cylinder and observed for phase separation. The ST in minutes was recorded as an average of three replicates per sample.

#### Particle size

The particle size (PS) distribution of the CNC-NS was analyzed using the nanoparticle size analyzer (M/s Cordouan Technologies®, France, scattering angle: 170°) based on the optical fiber dynamic light scattering and autocorrelation principles. The PS distribution was evaluated based on Dn 10%, Dn 50%, Dn 90% using the SBL® software, and the mean value was considered. Average of three replicates was reported for each sample.

#### Viscosity

The viscosity (V) of the suspension was estimated using a programmable rheometer (M/s Brookfield®, Model: LVDV-III, USA). The LV3 spindle was utilized to estimate viscosity at 100 rpm. About 50 ml suspension was taken for measurement and was replicated three times to obtain the average readings.

#### Surface morphology

Scanning electron microscopy (SEM) was performed using the setup (M/s Philips XL30 SEM, Netherlands) under an accelerating voltage of 10 kV and high vacuum mode. The samples were sonicated prior to placing them on the sample holder and air-dried for coating. The samples were then vacuum sputtered with a gold–palladium mixture to improve their conductivity and observed under SEM. The images were captured at three different magnifications (250×, 650×, and 2000×).

#### Surface tension

The surface tension (S) of the samples was measured by the pendant drop method, using a goniometer (M/s Rame-Hart Instrument, USA) coupled with image analysis software. The droplet shape hanging from the tip of a syringe was determined from the balance of forces, which included the surface tension of that liquid. The surface tension was calculated using the following mathematical equation:

2 $$\upgamma = \frac{\Delta \rho \times g \times Ro}{\beta }$$

where γ = surface tension, Δρ = difference in density between fluids at the interface, g = gravitational constant, Ro = radius of drop curvature at the apex, and β = shape factor. The surface tension was determined by fitting the shape of the drop (in a captured image) to the Young–Laplace equation. As per the experimental runs, different samples were prepared and filled in the syringe. The sample was dispensed through the needle forming a pendant drop of liquid hanging from the syringe. The surface tension test was conducted at 20 ± 1 °C and each measurement was repeated for five times.

#### Raman spectroscopy analysis

Raman Spectroscopy analysis of the samples was carried out using Antonpaar Cora 5000® Raman Spectrophotometer with a 1064 nm excitation wavelength. Spectrums were recorded with a spectral range from 300 to 2300 cm^−1^ with a resolution of 12 cm^−1^ and laser power of 450 mW. Spectrum analysis was carried out using inbuilt Cora Reader® software.

#### X-ray diffraction (XRD)

For the identification of the crystallinity phase of CNCs, elemental sulphur and optimized sample (CNC-NS), X-ray diffractometer (M/s Ultima IV, Rigaku, Japan with Cu Ka, k = 1.54 Å) irradiation was used.

#### Zeta potential

The zeta potential of the samples was measured using Nicomp™ 380 ZLS particle size analyser by electrophoretic light scattering principle. The electrodes were placed inside the sample cuvette to apply the required voltage and the scattered light due to Brownian motion of the particles are recorded, further converted into zeta potential, and reported in terms of mV. The scattering angle was kept at 90° for all analysis.

#### Thermo gravimetric analysis (TGA)

TGA analysis of the samples was carried out using the thermal analyser (M/s Netzsch TG 209 F3 Tarsus®). Thermal analysis was carried out using 30 ml/min flow rate of nitrogen atmosphere. Temperature range from 30 to 800 °C was maintained using 10 °C/min heating rate. The results were analysed using Netzsch Proteus® software.

#### Atomic force microscopy (AFM)

Atomic force microscopic (M/s Veeco®, Bruker, USA) was used to characterize the surface morphology of the CNCs. AFM height images of the samples were taken in the tapping mode at a frequency of 253 Hz using a silicon cantilever. The sample suspension was deposited onto the surface of freshly cleaved mica and dried under an IR lamp. The scan speed during imaging was kept at 512 and no filtering was done during the image acquisition.

#### Fourier transform infrared spectroscopy (FTIR)

Samples were subjected to attenuated total reflectance FTIR spectroscopy using a Shimadzu IR Prestige-21® spectrophotometer with diamond ATR attachment. Scanning was conducted from 4000 to 400 cm^−1^ with 49 repetitive scans averaged for each spectrum. The resolution was kept at 2 cm^−1^.

## Result and discussion

### CNCs characterization

**Figure 1:**
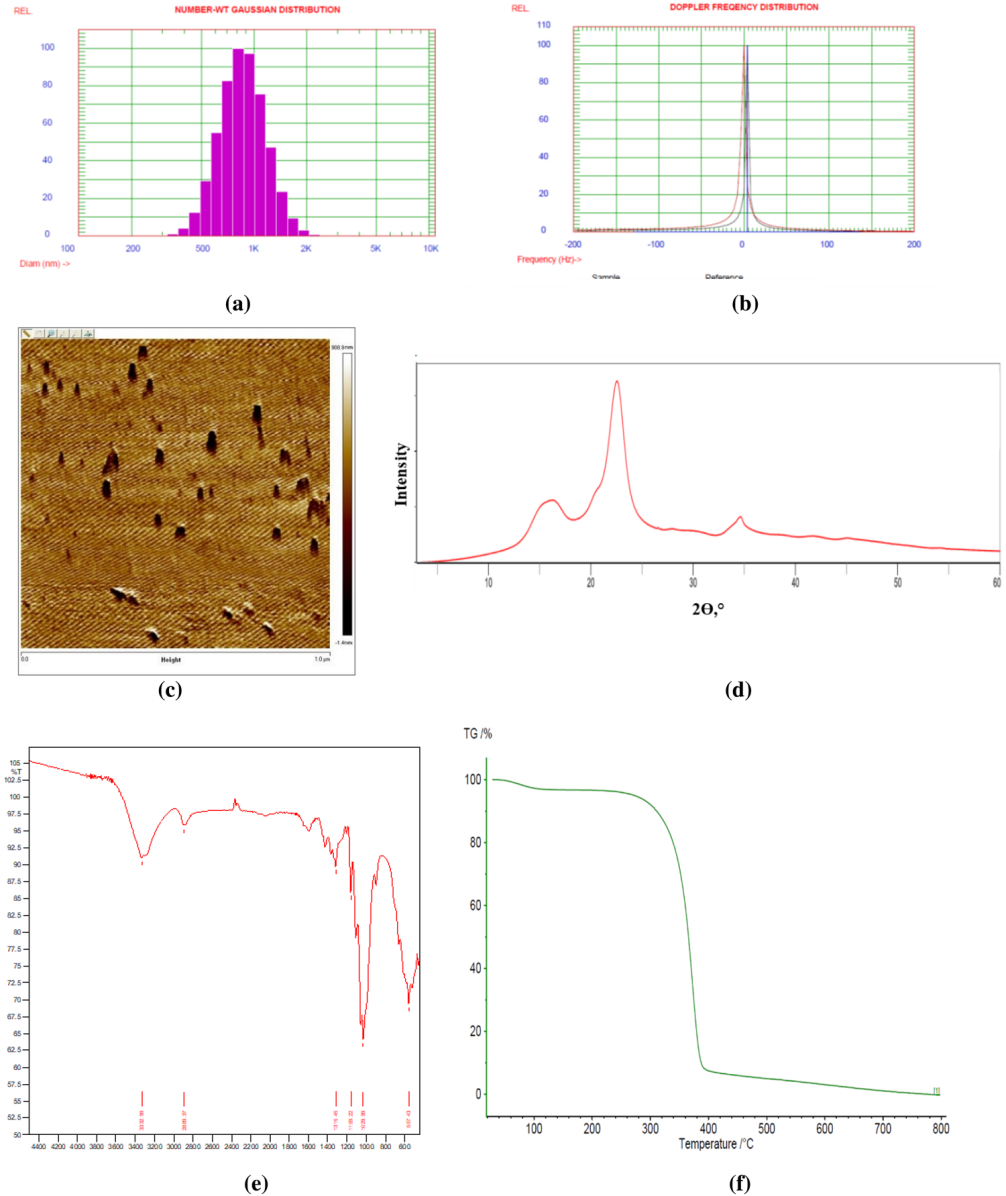
Characterization of CNCs (**a**) Particle size distribution showing the mean particle size in the range of 898 ± 175 nm (**b**) Zeta potential curve with the average value of 9.92 mV and viscosity of 0.933 cP (**c**) AFM micrographs confirming the size of CNCs as ≤ 900 nm (**d**) XRD pattern with diffraction angle (2θ) of 22.48° (**e**) FTIR curve of CNCs (**f**) TGA curve of CNCs.

The amount of CNCs present in the suspension after lyophilisation was estimated to be 5% (w/v). Thus, for the CNCs volume i.e. 25–75 ml used for the experiments, the CNCs concentration was ranged from 1.25 to 3.75%. The particle size was in the range of 898 ± 175 nm, and the zeta potential was 9.92 mV which has corroborated the stability of the CNCs suspension. The nano size range of the CNCs was further confirmed with the AFM analysis as shown in Fig. 1. The XR diffraction analysis of CNCs showed that there was intense peak at the diffraction angle (2θ) of 22.48° which was in accordance with the published literature. The FTIR data analysis showed the typical cellulose peaks of CNC, such as the stretching of the OH group at 3332 cm^−1^, and the stretching and deformation vibrations of the CH_2_ group in the glucose unit at 2897 cm^−1^. The TGA curve revealed that CNCs decomposition started at 300 °C and persisted until 400 °C.

### Model establishment and analysis

The moisture content of the CNC-NS was 93.92 ± 0.42 (wet basis). The data pertaining to the responses i.e. settling time, particle size, viscosity, and surface tension were determined and are mentioned in the design matrix obtained using RSM. The BBD matrix with corresponding values of the responses is presented in Table 2. The experimental data of the responses was most accurately fitted to a quadratic model as also confirmed by ANOVA. The coefficient of variation (CV) is defined as the ratio of the standard error of estimate to the mean value of the observed response and is a measure of the reproducibility of the model. A lower value of CV shows a high degree of accuracy and a good level of reliability of the experimental values. Lack of fit (LOF) significance is a good indication to predict model adequacy, and an insignificant LOF suggests that the model fits the experimental data satisfactorily. Adequate (Adeq) precision value denotes the signal to noise ratio, and a ratio greater than 4 is desirable. The ANOVA depicts that for all the responses, the adeq precision was above 4 indicating adequate signal for model fitting. The *p* values were the indication of the interaction pattern among the variables to check the significance of the coefficients. The coefficient with a lower ‘*p*’ value is highly significant. For simplification, insignificant terms from the second-order model equations for all responses were removed. The ANOVA results including the statistical parameters are presented for settling time (Table 3), particle size (Table 4), viscosity (Table 5), and surface tension (Table 6), respectively.

**Table 2:** Three factor three level BBD matrix and responses.

Run	Independent parameters			Responses			
	CNCs (A)	ES (B)	Passes (C)	Settling time (min)	Particle size (nm)	Viscosity (100 rpm)	Surface tension (N/m)
1	75 (1)	5 (1)	2 (0)	20.60**	500.41*	200.00	68.53
2	50 (0)	10 (0)	2 (0)	6.33	714.98	161.40	61.88
3	75 (1)	10 (0)	3 (1)	18.79	519.25	339.60	67.44
4	50 (0)	15 (1)	1 (− 1)	2.89	616.31	64.72	71.85
5	75 (1)	10 (0)	1 (− 1)	11.21	665.15	190.00	71.66
6	50 (0)	10 (0)	2 (0)	6.36	712.46	157.00	62.93
7	25 (− 1)	10 (0)	1 (− 1)	0.95	1432.62**	33.96	71.44
8	25 (− 1)	15 (1)	2 (0)	0.84*	1011.53	29.20*	72.26
9	50 (0)	10 (0)	2 (0)	6.22	713.95	154.60	65.63
10	75 (1)	15 (1)	2 (0)	7.29	614.76	420.60**	60.35*
11	25 (− 1)	5 (1)	2 (0)	1.33	1196.73	44.92	73.03
12	25 (− 1)	10 (0)	3 (1)	1.50	990.68	93.00	72.12
13	50 (0)	5 (1)	3 (1)	4.25	609.63	216.64	73.61**
14	50 (0)	10 (0)	2 (0)	6.25	715.50	155.32	62.38
15	50 (0)	5 (1)	1 (− 1)	3.15	699.00	83.60	71.93
16	50 (0)	10 (0)	2 (0)	6.31	725.32	158.26	63.95
17	50 (0)	15 (1)	3 (1)	5.35	542.22	162.25	72.35

*Corresponds to minimum value, **corresponds to maximum value.

**Table 3:** Analysis of variance with linear, quadratic and interaction effect of the independent variables on ST.

Source	Sum of squares	df	Mean square	F-value	*p* value
Model	488.22	9	54.25	12.73	0.0015
A	354.71	1	354.71	83.23	< 0.0001
B	21.00	1	21.00	4.93	0.0619
C	17.08	1	17.08	4.01	0.0854
A × B	41.09	1	41.09	9.64	0.0172
A × C	12.36	1	12.36	2.90	0.1324
B × C	0.46	1	0.462	0.11	0.7515
A^2^	30.96	1	30.96	7.26	0.0308
B^2^	9.36	1	9.36	2.20	0.1820
C^2^	3.36	1	3.36	0.789	0.4041
Residual	29.83	7	4.26		
Lack of fit	29.82	3	9.94	2985.03	< 0.0001
Pure error	0.013	4	0.003		
Cor total	518.05	16			

R-squared = 0.9424, Adjusted R-squared = 0.8684, Adeq Precision = 12.46, CV (%) = 32.02.

**Table 4:** Analysis of variance with linear, quadratic and interaction effect of the independent variables on particle size.

Source	Sum of squares	df	Mean square	F-value	*p* value
Model	9.928E + 05	9	1.103E + 05	29.63	< 0.0001
A	6.798E + 05	1	6.798E + 05	182.60	< 0.0001
B	6102.36	1	6102.36	1.64	0.2412
C	70556.46	1	70556.46	18.95	0.0033
A × B	22432.55	1	22432.55	6.03	0.0438
A × C	21909.92	1	21909.92	5.89	0.0457
B × C	58.37	1	58.37	0.016	0.9039
A^2^	1.680E + 05	1	1.680E + 05	45.14	0.0003
B^2^	30679.10	1	30679.10	8.24	0.0240
C^2^	860.08	1	860.08	0.23	0.6454
Residual	26058.85	7	3722.69		
Lack of fit	25954.94	3	8651.65	333.04	< 0.0001
Pure error	103.91	4	25.98		
Cor total	1.019E + 06	16			

R-squared = 0.9744, Adjusted R-squared = 0.9415, Adeq Precision = 18.58, CV (%) = 7.99.

**Table 5:** Analysis of variance with linear, quadratic and interaction effect of the independent variables on viscosity.

Source	Sum of squares	df	Mean square	F-value	*p* value
Model	1.591E + 05	9	17673.92	10.02	0.0031
A	1.126E + 05	1	1.126E + 05	63.84	< 0.0001
B	2165.15	1	2165.15	1.23	0.3045
C	24113.18	1	24113.18	13.67	0.0077
A × B	13961.79	1	13961.79	7.92	0.0260
A × C	2050.28	1	2050.28	1.16	0.3167
B × C	315.24	1	315.24	0.179	0.6851
A^2^	2496.67	1	2496.67	1.42	0.2729
B^2^	268.58	1	268.58	0.153	0.7080
C^2^	1293.42	1	1293.42	0.733	0.4202
Residual	12346.32	7	1763.76		
Lack of fit	12317.29	3	4105.76	565.71	< 0.0001
Pure error	29.03	4	7.26		
Cor total	1.714E + 05	16			

R-squared = 0.9280, Adjusted R-squared = 0.8354, Adeq Precision = 11.04, CV (%) = 26.79.

**Table 6:** Analysis of variance with linear, quadratic and interaction effect of the independent variables on surface tension.

Source	Sum of squares	df	Mean square	F-value	*p* value
Model	301.01	9	33.45	6.16	0.0128
A	54.44	1	54.44	10.03	0.0158
B	13.24	1	13.24	2.44	0.1625
C	0.2312	1	0.232	0.043	0.8424
A × B	13.73	1	13.73	2.53	0.1559
A × C	6.00	1	6.00	1.11	0.3280
B × C	0.3481	1	0.348	0.064	0.8074
A^2^	12.30	1	12.30	2.27	0.1760
B^2^	50.97	1	50.97	9.39	0.0182
C^2^	132.12	1	132.12	24.33	0.0017
Residual	38.02	7	5.43		
Lack of fit	29.18	3	9.73	4.40	0.0931
Pure error	8.84	4	2.21		
Cor total	339.03	16			

R-squared = 0.8879, Adjusted R-squared = 0.7437, Adeq Precision = 6.45, CV (%) = 3.41.

### Settling time

The minimum and maximum ST was observed as 0.84 and 20.60 min for experimental runs 8 and 1, respectively. It can be observed that a higher amount of ES (15 g) resulted in a lower ST, and simultaneously higher amount of CNCs (75 ml) yielded a higher ST. The cloudy appearance of the suspension was the probable outcome of the increasing CNCs percentage. The ANOVA for the response surface quadratic model of ST is shown in Table 3. The proposed model suggests that the amount of CNCs, ES, and NP had a significant effect (*p* < 0.05) on ST. The R^2^ = 0.9424 was not closer to the adjusted R^2^ of 0.8684, however, the model F-value of 12.73 implies that the model was significant. The *p*-values indicated that the linear terms of A (CNCs), B (ES), interactive term of AB (CNCs × ES), and quadratic term of A^2^ (CNCs) are significant (Table 3).

**Figure 2:**
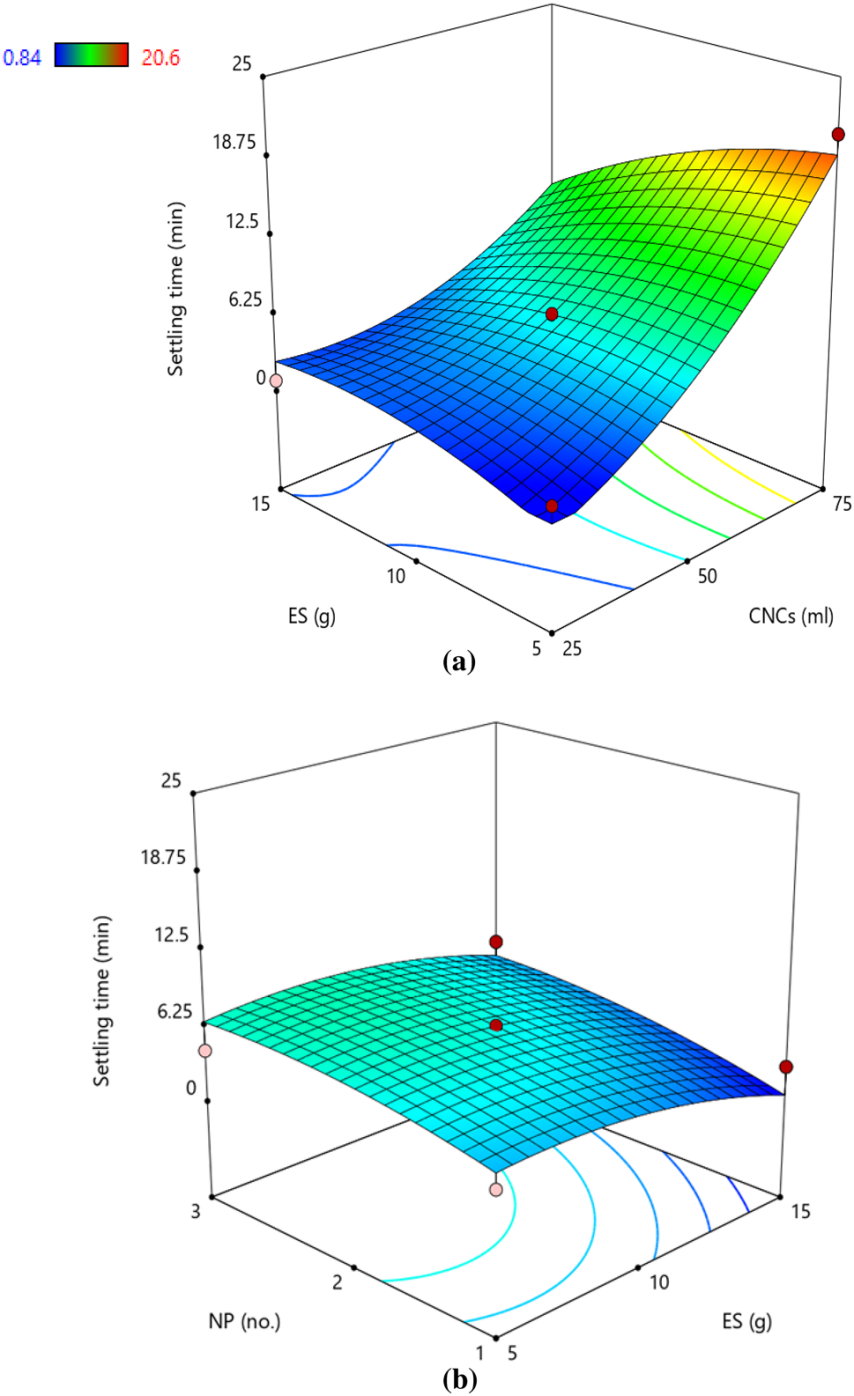
Response surface plot demonstrating the effect of (**a**) cellulose nanocrystals and elemental sulphur (**b**) cellulose nanocrystals and number of passes on settling time. The trend showed that increased proportion of CNCs resulted in increased ST, however the effect of ES and NP was insignificant. Colour pattern in the graph corresponds to the value of ST where blue (minimum), green (intermediate), yellow–red (maximum).

Figure 2 demonstrates the effect of the concentration of CNCs and ES on the ST of suspension. It was observed that the increased proportion of CNCs resulted in a profound increase in ST. Similarly, as the concentration of ES increased there was an insignificant increase in the ST as evident from Fig. 2a. Similar trend for the meagre increase in ST with the NP was also observed (Fig. 2b). The polynomial regression equations were generated in coded (Eq. 3) and actual terms (Eq. 4) and are mentioned below:

3 $${\mathbf{ST}} = 6.29 + 6.66{\text{A}} - \, 3.21{\text{AB}} + 2.71{\text{A}}^{2}$$

4 $${\mathbf{ST}} = - 9.83 - 0.052{\text{CNCs}} - 0.026{\text{CNCs}} \times {\text{ES}} + 0.004{\text{CNCs}}^{2}$$

### Particle size

**Figure 3:**
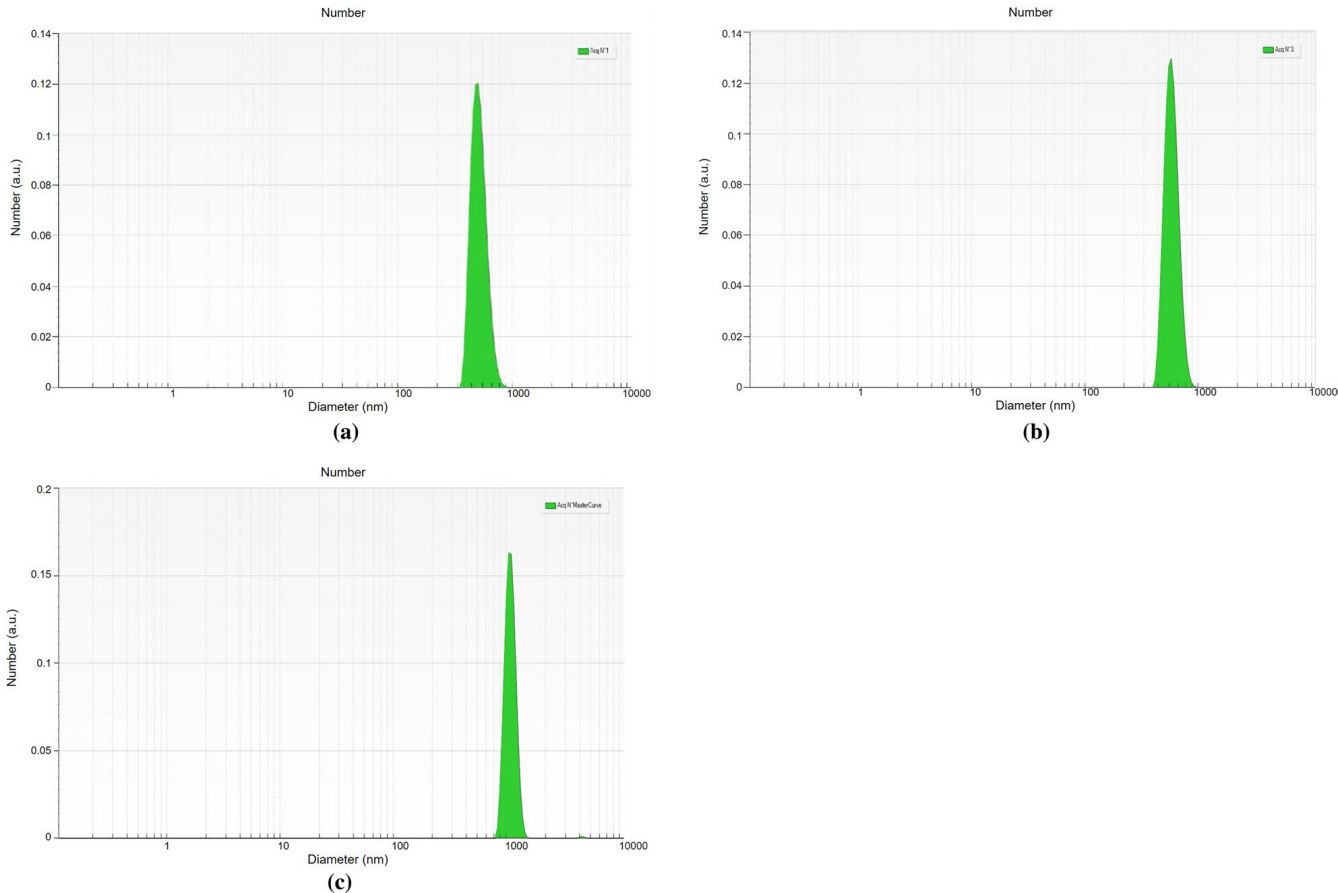
Particle size distribution graph generated using the SBL software (**a**) Run 3 (Mean size 519.25 ± 75.08 nm) (**b**) Run 6 (Mean size 712.46 ± 92.65 nm) (**c**) Run 8 (1011.53 ± 100.40 nm).

The average PS of the obtained suspensions was in the range of 500.41 nm and 1432.62 nm for experimental runs 1 and 7, respectively. The suspension with higher CNCs (75 ml) and minimum ES (5 g) passed twice in the homogenizer resulted in better size reduction indicating that the presence of CNCs might have assisted in the smooth mechanical shearing of the particles. Ren et al.^18^ illustrated that the high-pressure homogenizer treatments effectively reduced the dimension of the cellulose nanocrystals. The representative particle size distribution of the random experimental runs (3, 6, and 8) is shown in Fig. 3.

ANOVA for the response surface quadratic model of PS was presented in Table 4. The proposed model suggests that the A (CNCs), B (ES), and C (NP) had a non-significant effect on PS. The coefficient of determination value (R^2^ = 0.9744) indicates that the response model can explain 97% of the total variations which is lower than settling time. The model F-value of 29.63 implies there is a 7.48% chance that an F-value this large could occur due to noise. For the particle size response, the linear effect of A (CNCs), the interactive effect of AB (CNCs × ES), AC (CNCs × NP), and the quadratic effect of A (CNCs) and B (ES) were found to be significant model terms (Table 4). The lower CV value (7.99%) also confirmed a higher degree of accuracy and a good level of reliability of the experimental values.

**Figure 4:**
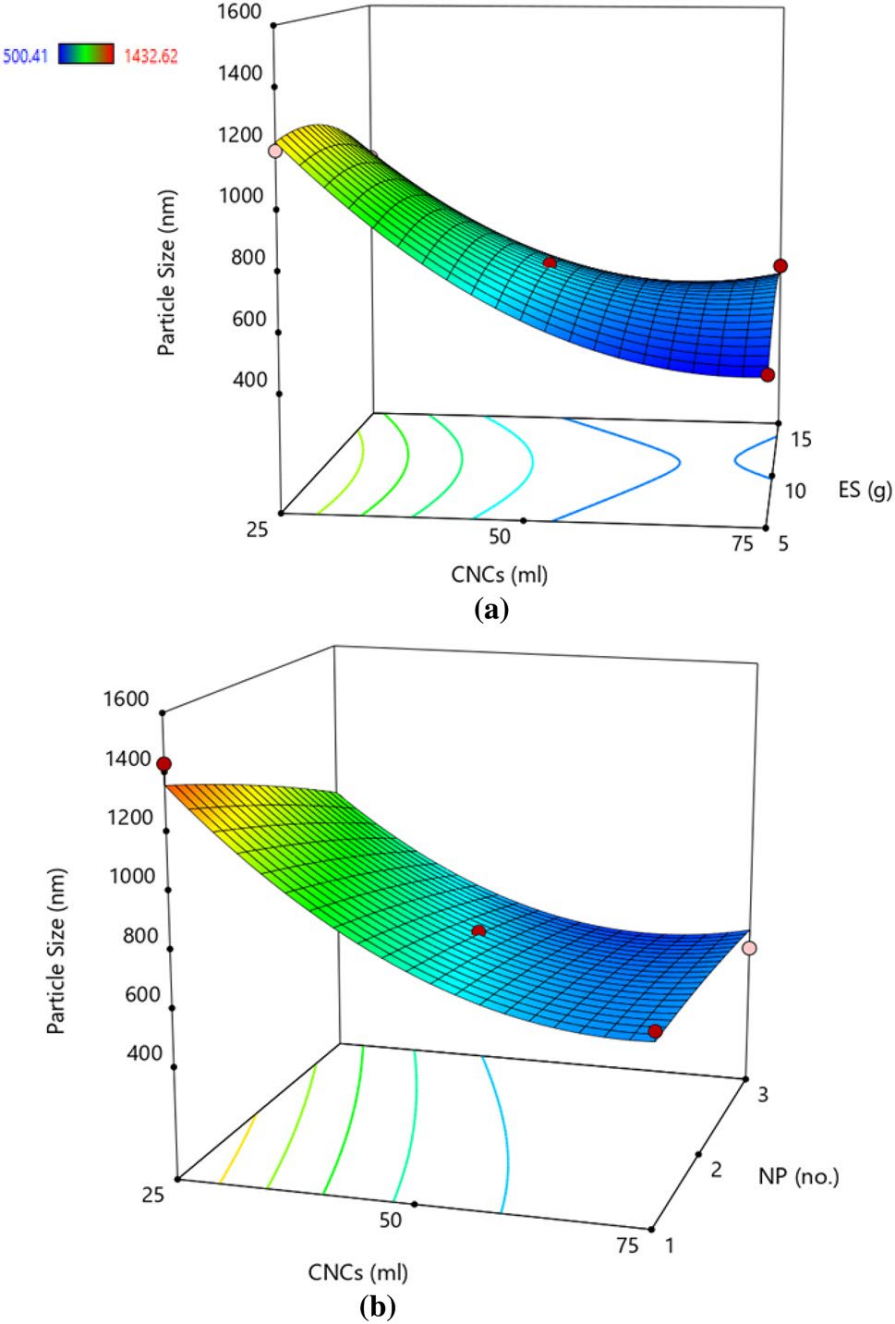
Response surface plot demonstrating the effect of (**a**) cellulose nanocrystals and elemental sulphur (**b**) cellulose nanocrystals and number of passes on particle size. The trend showed that increased CNCs resulted in finer particle size, the effect of higher ES was detrimental and the effect of NP was insignificant. Colour pattern in the graph corresponds to the value of PS where blue (minimum), green (intermediate), yellow–red (maximum).

The response surface plot showed that the increasing amount of CNCs has a desirable positive effect on the PS i.e. samples processed with higher CNCs concentration tend to have finer size. This was attributed to the binding property of CNCs resulted in an efficient homogenization for a stable CNCs–ES suspension. The increasing proportion of ES resulted in agglomeration of particles and thereby augmented hydrodynamic diameter of the particles (Fig. 4a). However, the effect of the NP in the homogenizer showed an insignificant effect on the particle size (Fig. 4b). Using the actual results, a polynomial regression equation was generated as mentioned below in coded (Eq. 5) and actual terms (Eq. 6).

5 $${\mathbf{PS}} = 716.44 - 291.50{\text{A}} - 93.91{\text{C}} + 74.89{\text{AB}} + 74.01{\text{AC}} + 199.78{\text{A}}^{2} - 85.36{\text{B}}^{2}$$

6 $${\mathbf{PS}} = 2553.87 - 55.54{\text{CNCs}} - 192.40{\text{NP}} + 0.60{\text{CNCs}} \times {\text{ES}} + 2.96{\text{CNCs}} \times {\text{NP}} + 0.32{\text{CNCs}}^{2} - 3.41{\text{ES}}^{2}$$

Wang et al.^15^ elucidated the effect of high-pressure homogenization on the particle size of nanocellulose. The authors could achieve the mean particle size of 610 nm size after 30 cycles in the homogenizer at 40 to 140 MPa pressure. Fu et al.^19^ in their study extracted nanocellulose from pineapple leaf using high-pressure homogenization and observed a reduction in the particle size of cellulose with increased working pressure until 100 MPa owing to shearing action, cavitation, and collision phenomenon. Operating at higher pressure resulted in increased particle size due to agglomeration among particles by virtue of the van der Waals forces.

### Viscosity

**Figure 5:**
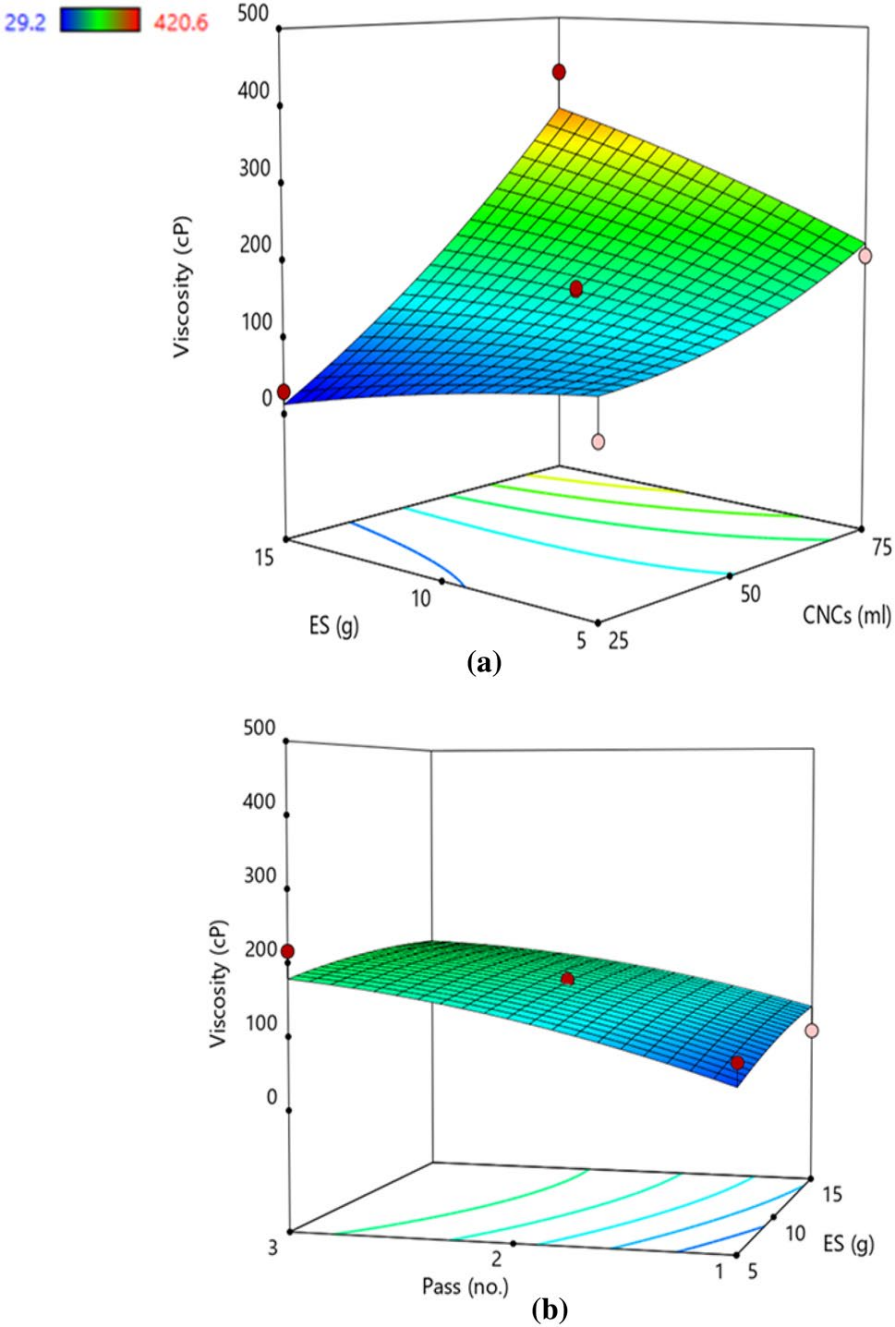
Response surface plot demonstrating the effect of (**a**) cellulose nanocrystals and elemental sulphur (**b**) elemental sulphur and number of passes on viscosity. The trend showed that increased CNCs and higher NP attributed to enhanced viscosity, however the presence of higher ES resulted in reduced viscosity. Colour pattern in the graph corresponds to the value of viscosity where blue (minimum), green (intermediate), yellow–red (maximum).

The viscosity values were recorded as 29.20 cP (minimum) and 420.60 cP (maximum), for experimental runs 8 and 10, respectively. This variation may be attributed to the rate of work required to align the suspension containing higher CNCs (75 ml) and correspondingly lower viscosity for the sample with lesser CNCs (25 ml). Enhanced dispersion of the CNCs and ES particles in the suspension can be attributed to the higher CNCs. The ANOVA for the response surface quadratic model of viscosity was presented in Table 5. Similar to particle size, the proposed model suggests that the amount of CNCs, ES, and NP had a significant effect on viscosity. Here, the model shows that the coefficient of determination value (R^2^ = 0.9280) indicated that the viscosity response model can explain about 90% of the total variations which is higher than settling time and particle size responses. The model F-value of 10.02 implies the model is significant. In this case, the linear term of A (CNCs) and the interactive term AB (CNC × ES) are significant model terms. It was evident that the correlation of viscosity with CNCs and the number of passes was positive (Fig. 5a). The presence of a higher amount of ES resulted in slightly declining values of viscosity (Fig. 5b). Using the actual results, a polynomial regression equation was generated as mentioned below in coded (Eq. 7) and actual terms (Eq. 8).

7 $${\mathbf{V}} = 157.32 + 118.64{\text{A}} + 54.90{\text{C}} + 59.08{\text{AB}}$$

8 $${\mathbf{V}} = 64.05 - 5.68{\text{CNCs}} + 97.48{\text{NP}} + 0.47{\text{CNCs}} \times {\text{ES}}$$

Peng and Via^20^ reported the effect of different pre-treatments on the viscosity of CNCs and observed that under the same shear stress, magnetic stirred CNCs had the highest viscosity than homogenized and sonicated CNCs samples.

### Surface tension

Surface tension has a linear correlation with the particle size of suspensions owing to the Van der Waals force between particles at the liquid/gas interface which increases surface free energy and thereby surface tension. The surface tension of the CNCs-NS suspensions was 60.35 N/m (minimum) and 73.61 N/m (maximum) for experimental runs 10 and 13, respectively. The ANOVA for the response surface quadratic model of surface tension is shown in Table 6. The proposed model suggests that the amount of coefficient of A (CNCs), B (ES), and C (NP) had a significant effect on S. Here, the coefficient of determination, R^2^ = 0.8879 indicated that the surface tension response model only can explain about 89% of the total variations which is lower than settling time, particle size, and viscosity. The model F-value of 6.16 implies the model is significant. In this case, the linear term of A (CNCs), quadratic terms of B (ES), and C (NP) are observed to be significant model terms.

**Figure 6:**
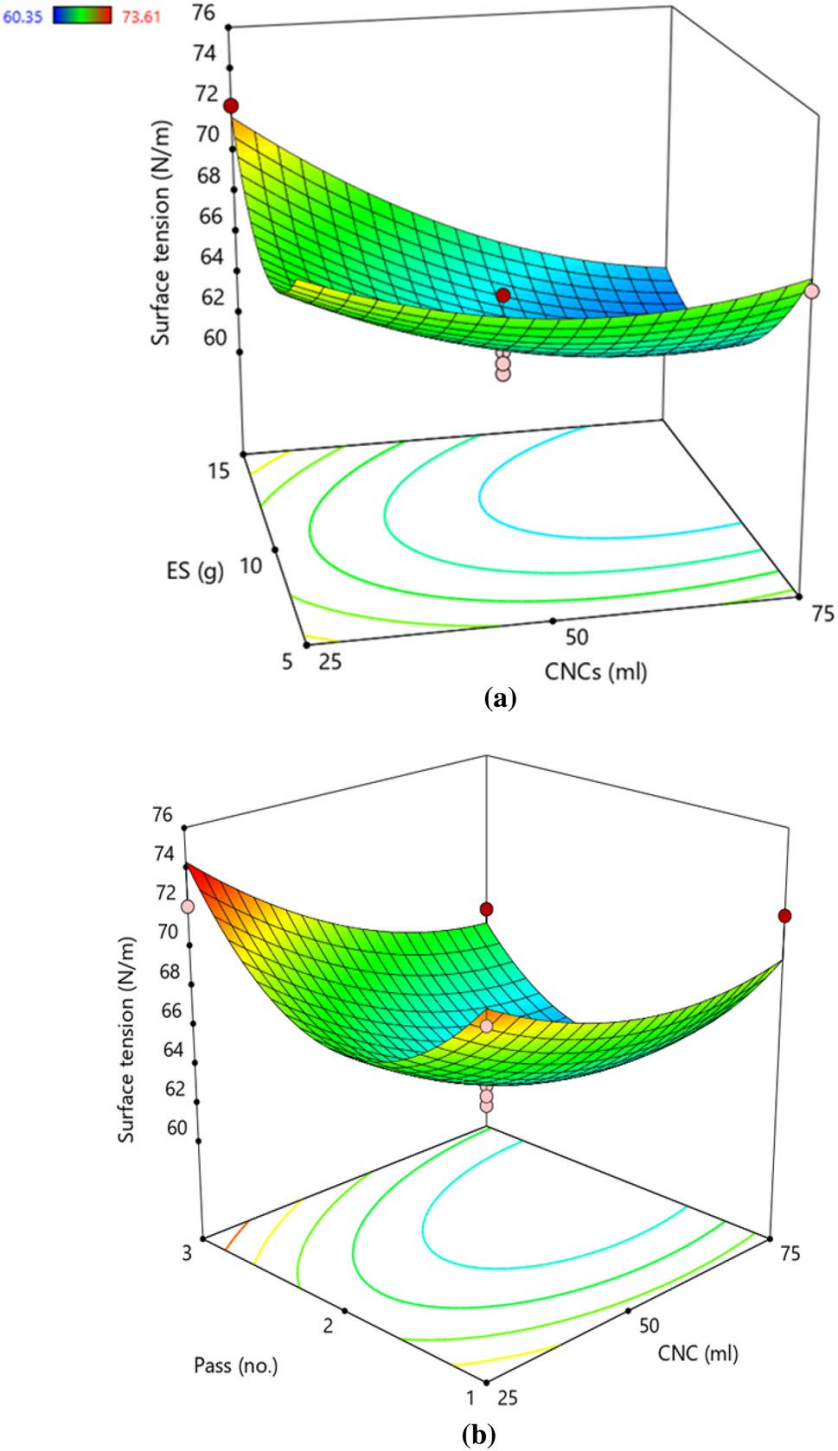
Response surface plot demonstrating the effect of (**a**) cellulose nanocrystals and elemental sulphur (**b**) elemental sulphur and number of passes on surface tension. The trend showed that ES and CNCs had insignificant effect on S, however, the S values initially decreased and further increased with NP. Colour pattern in the graph corresponds to the value of viscosity where blue (minimum), green (intermediate), yellow–red (maximum).

The response surface for the combined effect of the CNCs and ES (Fig. 6a) and ES and NP (Fig. 6b) are demonstrated. It can be observed from the contour graphs that the amount of ES and CNCs has an insignificant effect on S. The S values decreased initially with NP (1–2) and again increased at NP values from 2 to 3. Using the actual results, a polynomial regression equation was generated as mentioned below in coded (Eq. 9) and actual terms (Eq. 10).

9 $${\mathbf{S}} = 63.35 - 2.61{\text{A}} + 3.48{\text{B}}^{2} + 5.60{\text{C}}^{2}$$

10 $${\mathbf{S}} = 101.16 - 0.13{\text{CNCs}} + 0.14{\text{ES}}^{2} + 5.60{\text{NP}}^{2}$$

Bhuiyan et al.^21^ reported that the surface tension of the selected nanofluids increased with an increase in concentration and particle size. The reported trend was attributed to the increasing Van der Waals force between the accumulated particles at the liquid–gas interface, increasing the surface energy and thereby increasing surface tension. Mahendra et al.^22^ in their study reported the utilization of nanocellulose and NFC as reinforcing and compatibilizing agent of polypropylene/cyclic natural rubber blend. The results authenticated that the increase in nanocellulose loading in the blends has improved the interphase surface tension. Ledyastuti et al.^23^ demonstrated that the addition of nanocellulose slightly decreases the water–oil interface tension, further substantiating the role of nanocellulose as an emulsifier in the water–oil interface.

### Optimization

The numerical optimization technique was adopted to identify the optimized process parameters. The criteria goals for independent variables and process responses were kept within range. The optimization procedure yielded about 100 solutions and the solution with the highest desirability was obtained as, 47 g (CNCs), 8 g (ES), and 2 (NP) with the corresponding values of responses were 4.90 min (ST), 809 nm (PS), 107.98 cP (Viscosity) and 65.73 N/m (S), respectively.

### Scanning electron microscopy

**Figure 7:**
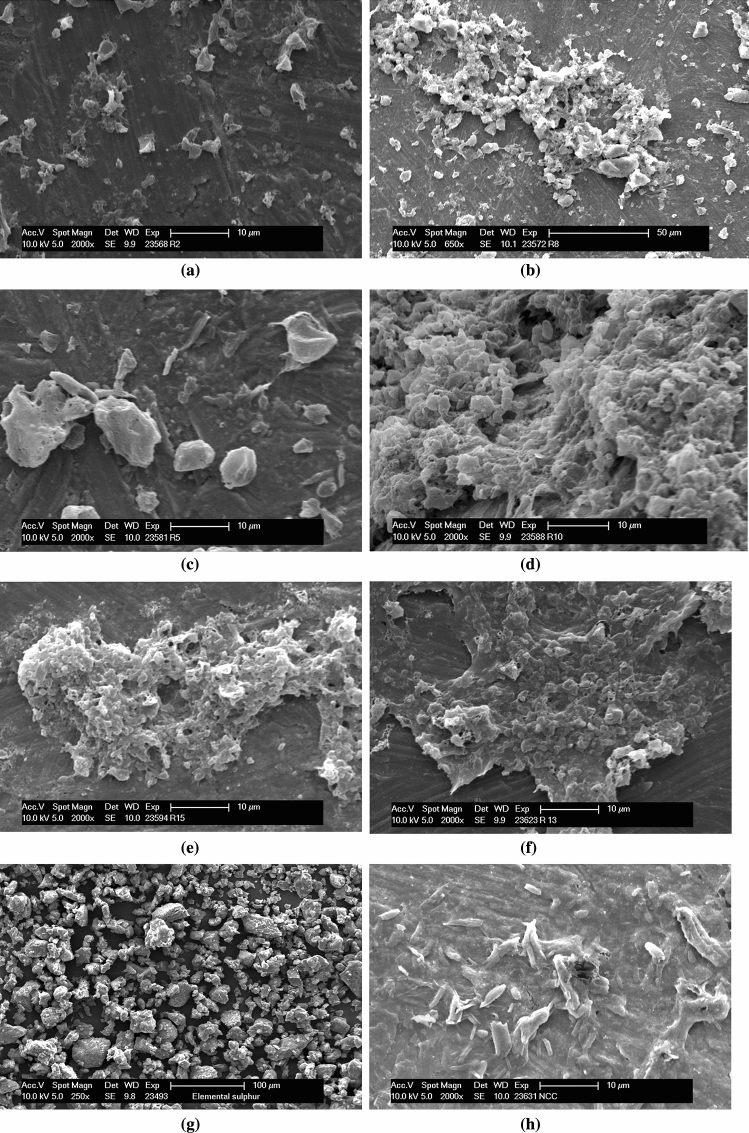
SEM micrographs of the samples (**a**) Run 2 (**b**) Run 8 (**c**) Run 5 (**d**) Run 10 (**e**) Run 15 (**f**) Run 13 (**g**) elemental sulphur (**h**) CNCs.

The morphological characterization of all 17 samples was performed. The microstructure analysis of selected experimental runs is illustrated in Fig. 7. It was observed that a higher number of passes in the homogenizer resulted in adequate mixing/bonding/deposition of ES with CNCs. The sample with a lesser amount of CNCs (25 ml) with the highest ES (15 g) and 2 passes showed scattered and less cohesive mixing among the particles (Fig. 7b). With a single pass, there was insignificant size reduction with minimum mixing of ES with CNCs (Fig. 7c) Also, the higher concentration of CNCs in the sample also resulted in the cumulative deposition of ES on cotton fibrils (Fig. 7d). Individual particles of ES having varied diameter in the range of micron size were observed (Fig. 7g). The SEM image of CNCs depicts the inherent fibrous structure with significant variation in fibre length and diameter (Fig. 7h).

### Raman analysis

**Figure 8:**
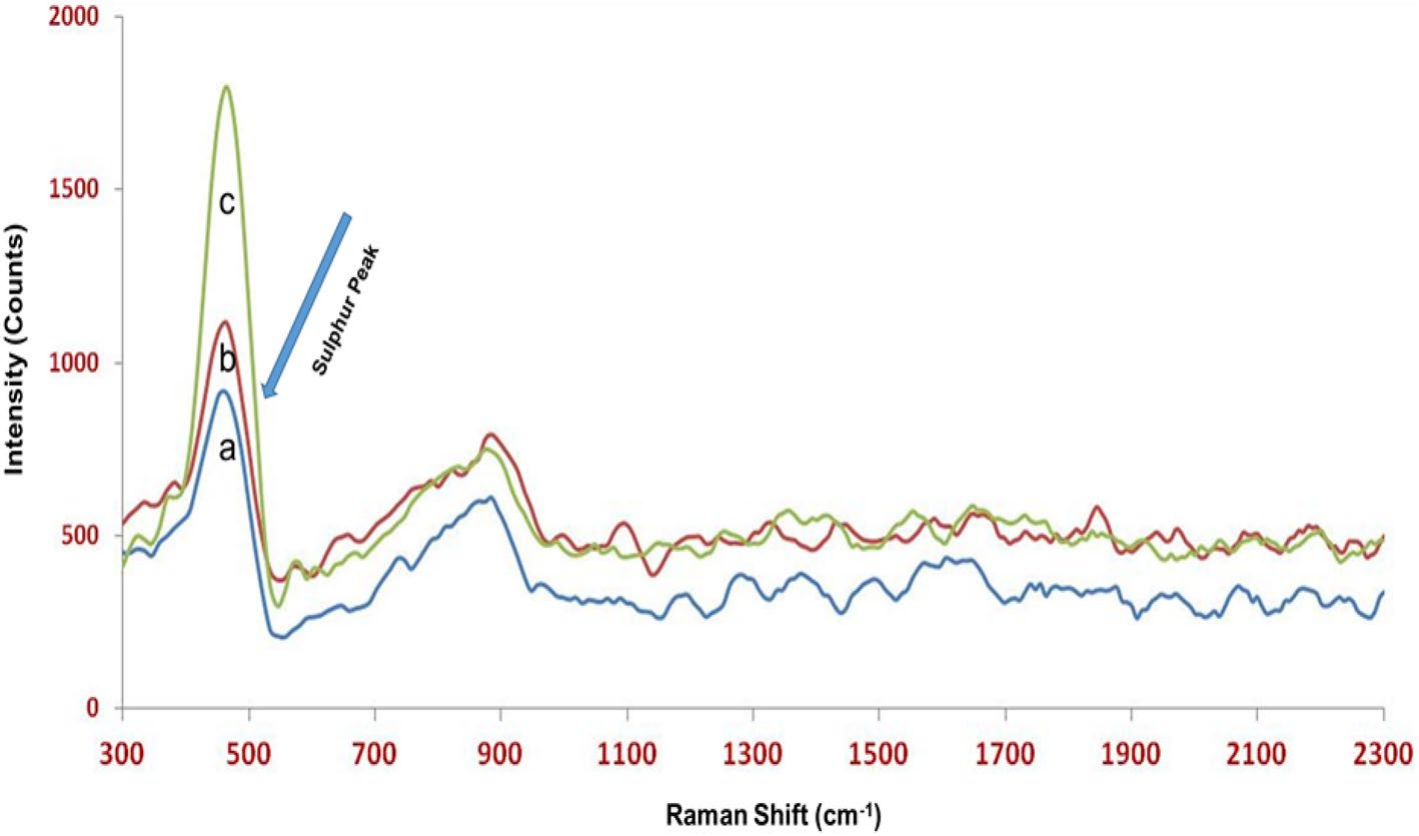
Raman spectrum of (**a**) Run 1 (**b**) Run 2 (**c**) Run 8.

Raman spectroscopic techniques are increasingly being used to investigate various cellulose nanocomposites due to the numerous advantages of this technique^24^. In this work, Raman spectroscopy was used as an additional characterization tool to precisely confirm the presence of sulphur in the cellulose matrix. In the spectra shown in Fig. 8, a clear non-cellulose band was detected at the Raman shift of 465 cm^−1^. This band is likely to be due to the –S– peaks present in the cellulose matrix because sulphur groups are known to contribute strong intense peaks at 460–480 cm^−1^ Raman shift^25,26^. It was observed that peak intensity was directly proportional to the quantity of sulphur present in CNC-NS. Additionally, promptness of cellulose peaks was hindered in the CNC-NS except in the region of 880–890 cm^−1^ due to highly intense sulphur peaks.

### X-ray diffraction

**Figure 9:**
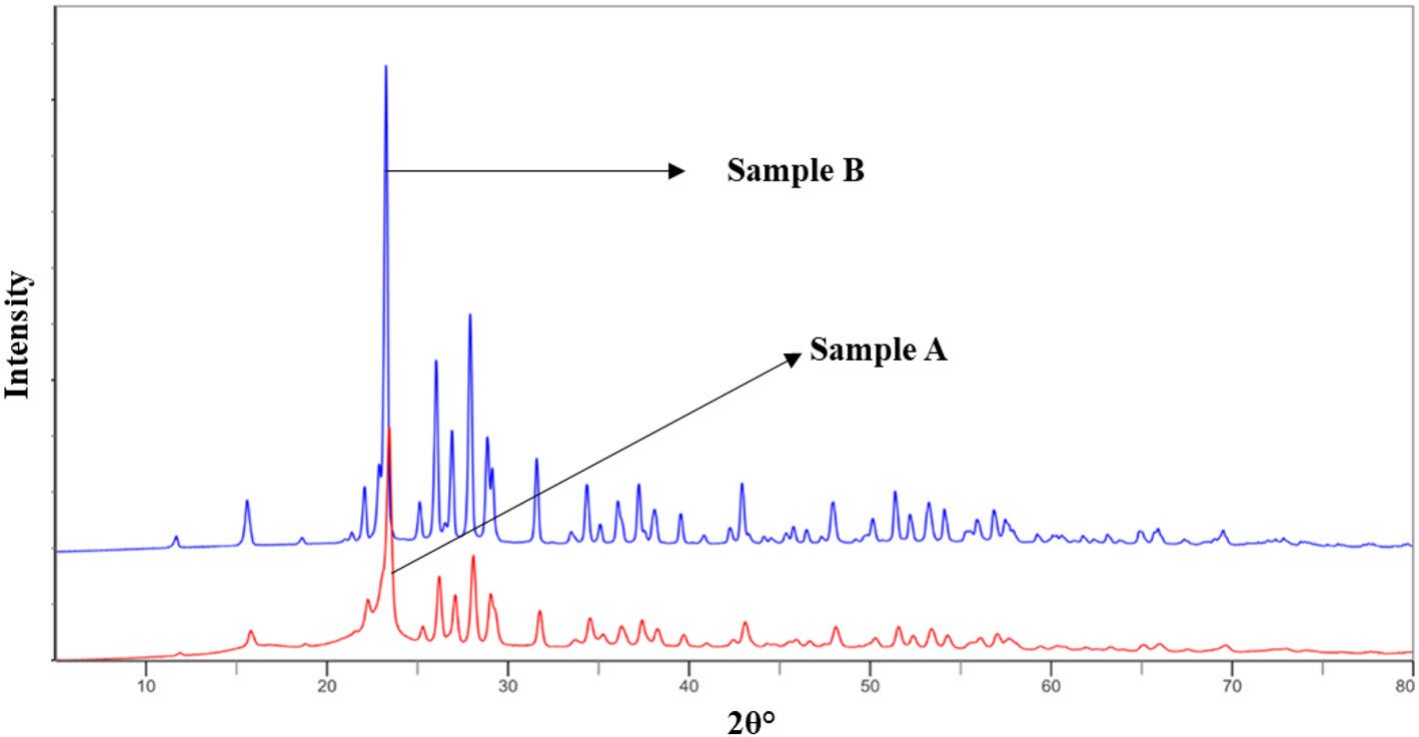
XRD graph of (**i**) sample A (optimized CNC-NS sample) (**ii**) sample B (elemental sulphur).

The crystallographic structure of a material can be known using XRD analysis. A XRD graph of sulphur crystals (sample B) and semicrystalline cellulose nanocrystals stabilized nanosulphur (Sample A) was presented in Fig. 9. In sample B, the 2θ peaks at 15.4°, 22°, 23.5°, 25.5°, 26.3, 27.6°, 28.5°, 31.5°, 33.5°, 37.5°, 38°, 44.01°, 47.01°, and 51.7° were attributed to the crystal planes of sulphur at 113, 220, 222, 040, 313, 044, 422, 319, 515, and 266, respectively. Similar peaks of sulphur crystals have been reported^27,28^. Cellulose I is a combination of two separate crystalline forms, cellulose I_α_ and cellulose I_β_. For the XRD pattern of cellulose I, the well-known diffraction peaks at 14.9°, 16.5°, 20.5°, 22.7°, and 34.5° are assigned to the typical reflection planes of cellulose I: 110, 110, 012, 200, and 004^29^. The diffraction peaks of nanocellulose indicated that the post-sulfation treatments did not destroy or convert the inherent crystalline structure of nanocrystals. The CNC-NS graph showed the peak shifting and peak overlap due to treatment with sulphur. The 2θ peaks at 12.1°, 15.4°, 22.7°, 31.5°, and 34.5° were showing the presence of cellulose I_α_ and cellulose I_β_^30^. Other diffraction peaks of nano crystalline cellulose overlap with sulphur crystal XRD peaks, which authenticates the presence of ES in CNCs.

### Zeta potential

**Figure 10:**
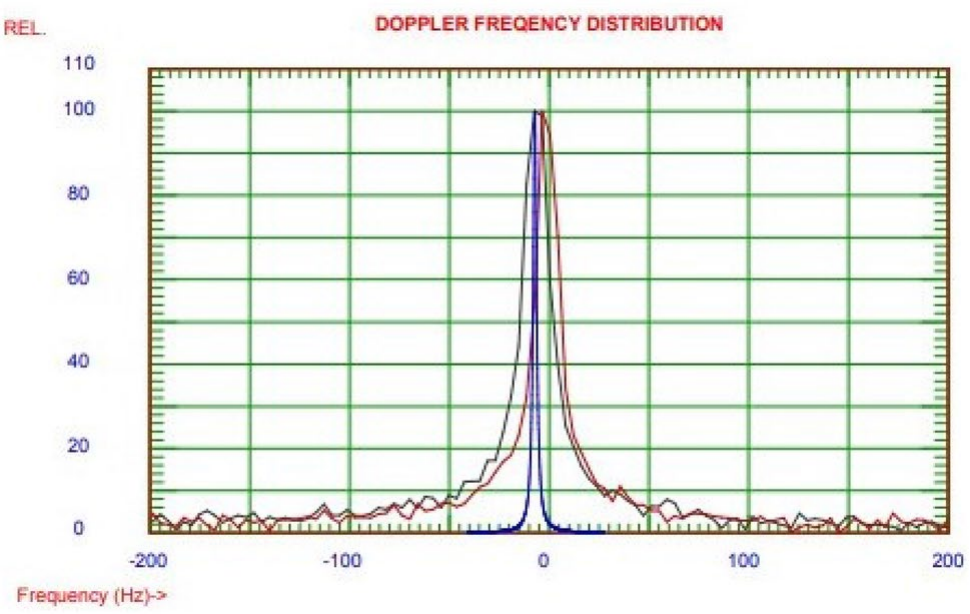
Zeta potential analysis of the optimized CNC-NS sample.

The zeta potential of the optimized CNC-ES sample was performed and the curve is shown in the Fig. 10. The observed zeta potential value was − 17.6 mV which indicated its strong stability as aqueous suspension. The findings were in agreement with previous studies which also reported negative zeta potential of sulphur nanoparticles. Baloch et al.^31^ synthesized the sulphur nano particles from *Citrus limon* leaves and reported the zeta potential value of − 12.32 mV. Also, Paralikar and Rai^32^ reported the zeta potential values (− 7.12 to − 34.1 mV) of sulphur nanoparticles synthesized using different plant extracts.

### Thermo gravimetric analysis (TGA)

**Figure 11:**
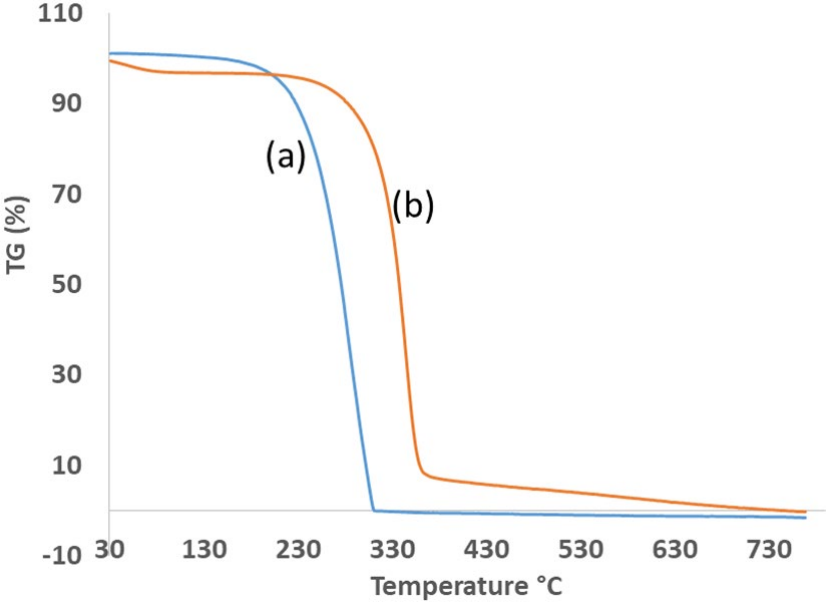
TGA graph of (**a**) elemental sulphur (**b**) optimized sample CNC-NS.

The TGA graph of elemental sulphur and the optimized CNC-NS sample is presented in Fig. 11. In Fig. 11a, the TGA graph of elemental sulphur exhibits a two-stage weight loss phenomenon. In the initial stage, a negligible weight loss is observed in the temperature range of 30–210 °C, which can be attributed to the evaporation of moisture. This is followed by the second stage, where the predominant weight loss, accounting for up to 99%, occurs between 210 and 315 °C. This significant weight loss can be attributed to the melting and subsequent evaporation of sulphur. In Fig. 11b, the TGA graph of the optimized CNC-NS sample illustrates weight loss occurring in three distinct stages. In the first stage, there is an 8% weight loss observed in the temperature range of 30–260 °C, which can be attributed to the moisture content within the cellulose molecules. The major weight loss, accounting for up to 85%, is observed between 260 and 380 °C. This substantial weight loss is primarily due to the degradation of treated nanocellulose and evaporation of sulphur. It was worth noting that the presence of sulphur catalyses the degradation of cellulose, leading to a more extensive degradation profile up to 380 °C, compared to the pure CNC, which typically exhibits degradation up to 400 °C.

### Fourier transform infrared spectroscopy

**Figure 12:**
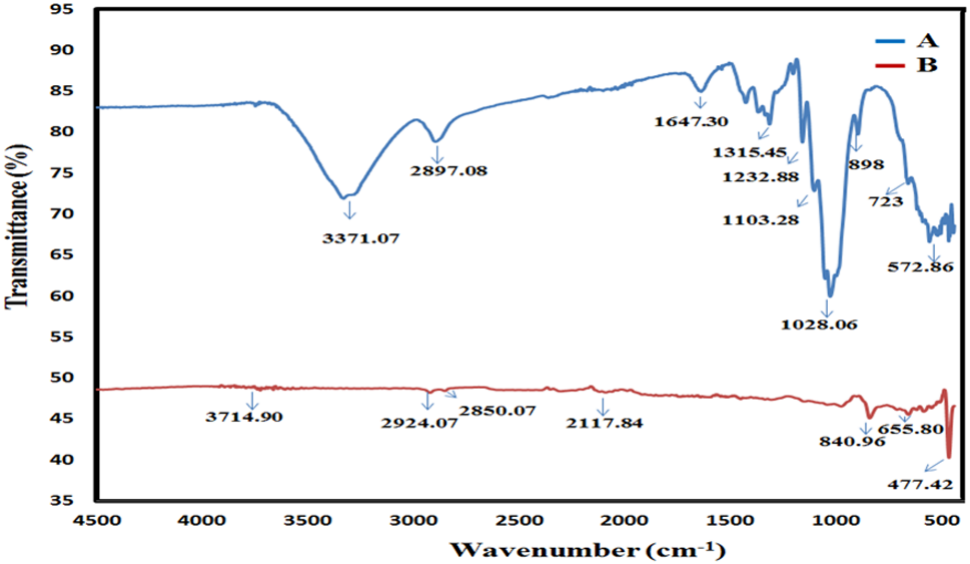
FTIR spectrum of (**A**) optimized sample CNC-NS (**B**) elemental sulphur.

FTIR spectrum of elemental sulphur (sample B) and nanocellulose-semicrystalline composite (Sample A) is presented in Fig. 12. In Fig. 12B, the transmittance band of sulphur crystal shows all characteristics peak at 477.42 cm^−1^, 655.80 cm^−1^, 840.96 cm^−1^ as reported earlier^33^. In Fig. 12A, the stretching of the hydroxyl group is attributed to the transmittance band at 3371.07 cm^−1^, and the stretching and deformation vibrations of the CH_2_ group in the glucose unit of CNC are assigned to the band at 2897.08 cm^1^. Other transmittance bands at 1647.20 cm^−1^, 1315.45 cm^−1^, and 1103.28 cm^−1^ are attributed to OH bending of absorbed water, C–H bending in the glucose unit, and C–O–C asymmetric stretching in β-glucosidic linkage respectively. The C–O group of secondary alcohols and ether functionalities found in the glucose chain backbone are attributed to the signal at 1028 cm^−1^. The strong peak of sulphur i.e. 840.96 cm^−1^ got merged with major peak of cellulose at 1028 cm^−1^ which confirms the presence of sulphur with the cellulose.

## Conclusions

This work was intended to prepare a stable nanosulphur formulation using CNCs as a stabilizer by a high-pressure homogenization process, optimized using RSM. The numerical optimization technique of RSM resulted in optimized process parameters i.e., 46 ml CNCs, 8 g ES, and 2 NP at 2100 kg/cm^2^ working pressure, to achieve a stable nanosulphur suspension. At the optimized conditions, the responses viz. settling time (4.90 min), average particle size (809 nm), viscosity (107.98 cP), and surface tension (65.73 N/m) were recorded. The results confirmed that an increase in the CNCs concentration resulted in stable suspension. The findings from this study emphasized for further application of CNCs as a stabilizer against chemical surfactants. The need of the developed nanosulphur formulation is consistent with the growing interest in capitalizing natural and eco-friendly products for their potential application in diversified industries including paper, textile, fertilizer, and agriculture.

## Data Availability

All data generated or analysed during this study are included in this published article.

## Abbreviations

RSM: Response surface methodology
NS: Nanosulphur
ES: Elemental sulphur
CNCs: Cellulose nanocrystals
CNC-NS: Cellulose nanocrystals stabilized nanosulphur
NFC: Nano fibrillated cellulose
BBD: Box–Behnken design
NP: Number of passes
SEM: Scanning electron microscopy
cm: Centi metre
ST: Settling time
S: Surface tension
V: Viscosity
Dn: Number average diameter
cP: Centi poise
ml: Milli litre
g: Gram
N/m: Newton per metre
ANOVA: Analysis of variance
PS: Particle size
CV: Coefficient of variation
kV: kilo volt
nm: Nano metre
mW: Milli watt
R^2^: Coefficient of determination
LOF: Lack of fit
Adeq: Adequate
XRD: X-ray diffraction
